# Development of a nurse-led clinical pathway to prevent delirium in older adults in general hospital wards: a realist review protocol

**DOI:** 10.1136/bmjopen-2025-105801

**Published:** 2025-09-15

**Authors:** Jonas Hoch, Martin Müller, Inga Unger, Anika Mitzkat, Natascha-Elisabeth Denninger

**Affiliations:** 1 Department of Primary Care and Health Services Research, Nursing Science and Interprofessional Care, Heidelberg University Medical Faculty Heidelberg, Heidelberg, Germany; 2 Heidelberg University Hospital Department of Internal Medicine, Heidelberg, Germany

**Keywords:** Implementation Science, Delirium, Hospitals, GERIATRIC MEDICINE

## Abstract

**Abstract:**

**Introduction:**

Delirium is a critical and complex neuropsychiatric syndrome that significantly affects older adults in general hospital wards. Although multicomponent interventions have been shown to be effective in preventing delirium, the consistent implementation remains a challenge. Also, to manage the complex pathway of patients from admission to discharge in hospital, the involvement of the nursing staff is essential. Developing a nurse-led clinical pathway for delirium prevention could provide a structured approach to improving care quality. For intervention development taking account of the complexity of the clinical environment, the UK Medical Research Council framework is frequently used. A core element of this framework is mapping a programme theory that explains how, for whom and in what circumstances an intervention may work. The realist review methodology is well suited to uncovering the underlying mechanisms, contexts and outcomes of interventions, translating these into a programme theory.

The aim of this realist review is to develop a programme theory for a nurse-led clinical pathway to prevent delirium in older adults aged 65 years or older in general hospital wards and to identify strategies to support its effective implementation.

**Methods and analysis:**

The realist review is based on the methodical framework developed by Pawson *et al* and further adapted by Rycroft-Malone *et al* and the reporting will follow the Realist And MEta-narrative Evidence Syntheses: Evolving Standards guidelines. The process comprises four steps: (1) defining the review scope; (2) systematically searching for and appraising the evidence; (3) extracting and synthesising findings and (4) developing a narrative synthesis. Interest holders, including clinical and academic experts, will be actively involved as an expert reference group to inform and refine the programme theory. The final programme theory will be presented in Context-Mechanism-Outcome configurations and the Implementation Research Logic Model.

**Ethics and dissemination:**

Since no data are collected as part of the review, ethical approval is not required. Findings will be disseminated through academic conferences and publication in a peer-reviewed journal.

**Registration:**

This protocol has been registered at Open Science Framework (https://doi.org/10.17605/OSF.IO/7EPTF).

**STRENGTHS AND LIMITATIONS OF THIS STUDY**

- The initial programme theory developed in this review may help to guide the design of future complex interventions for delirium prevention.
- The programme theory will be developed through iterative synthesis of diverse data sources.
- Interest holder engagement is integrated throughout the review to inform scope and interpretation.
- Quality appraisal focuses on relevance and rigour rather than risk of bias, which may affect comparability with traditional systematic reviews.

## Introduction

 Delirium is a critical and complex neuropsychiatric syndrome that is characterised by abrupt and sudden changes in various cognitive functions such as attention, consciousness, cognitive abilities or perception.^1^ In acute hospital care, it is a common complication for older adults aged 65 years or older,^2 3^ associated with a loss of quality of life, prolonged hospital stay, an increased risk for persistent cognitive impairment and need for long-term care. Delirium is also a financial burden for the healthcare system and associated with increased and unexpected workload, in particular for nurses.^4^,^7^ While nurses have a crucial role in preventing, recognising, communicating, monitoring and managing delirium, current research showed an insufficient utilisation of appropriate interventions in daily routine.^8^ Reasons are based on the organisational environment, individual prioritisation of care measures to balance workload, lack of delirium knowledge and a lack of clinical skills.^9 10^

In contrast, evidence highlighted that multicomponent, non-pharmacological interventions, targeting various risk factors for delirium like re-orientation, cognitive stimulation and sleep hygiene, were associated with a 27–54% reduction in delirium incidence among older people in general hospital wards.^11^,^13^ There is also evidence that nursing skill influences the quality of care, particularly through nurse-led non-pharmacological delirium interventions that are associated with reduced mortality.^14 15^ For example, the Hospital Elder Life Program^16^ is a widely used and investigated intervention in the hospital setting.^17^ Despite promising evidence, implementation of comprehensive nurse-led delirium prevention programmes in acute hospital care is scarce.^18^

A possible approach to implementing such complex intervention programmes are clinical pathways (CPWs).^19^ Defined as structured multidisciplinary care plans that detail essential steps in the management of a specific condition as a criteria-based progression, CPWs offer a strategy for quality improvement by enhancing patient outcomes, streamlining the care process and optimising resource utilisation.^19 20^ Positive effects of CPWs, especially the association with reduced in-hospital complications, reduced length of stay and improved documentation have been demonstrated; also, there were signs for reduced hospital costs, with further research needed.^21 22^

Despite the integral collaboration of different professions and disciplines, a delirium pathway should be nurse-led, since the recognition of patients’ immediate and often subtle behavioural or cognitive changes requires continuous presence and clinical judgement. Nurses are uniquely positioned to identify the fluctuating course of delirium through systematic observation, bedside assessment and the integration of contextual and biographical knowledge. These competencies are essential for timely detection, escalation and intervention core elements of effective delirium management. Therefore, the leadership of a delirium CPW should be rooted in nursing expertise, while embedded in an interprofessional framework, for example, if aspects like deprescribing of inappropriate medication or specific mobility training might be included.^14 23^ So, a recent study reported a reduction in delirium rates by using a CPW among older adults in geriatric trauma departments.^24^ However, research on CPWs for delirium prevention and management in general ward settings, especially those emphasising a central role for nurses, is missing.^11^ The UK Medical Research Council (MRC) framework is frequently used to address the described complexity of interventions in a clinical environment.^25^ The framework defines four phases (development or identification of interventions, feasibility, evaluation and implementation), underpinning core elements like programme theory, interest-holder perspective and context.^26^ The framework’s key strength is the research focus on the real-life conditions for implementing complex interventions like acceptability, implementability, cost effectiveness, scalability and transferability across contexts, then only based on effectiveness.^26^

**Figure 1:**
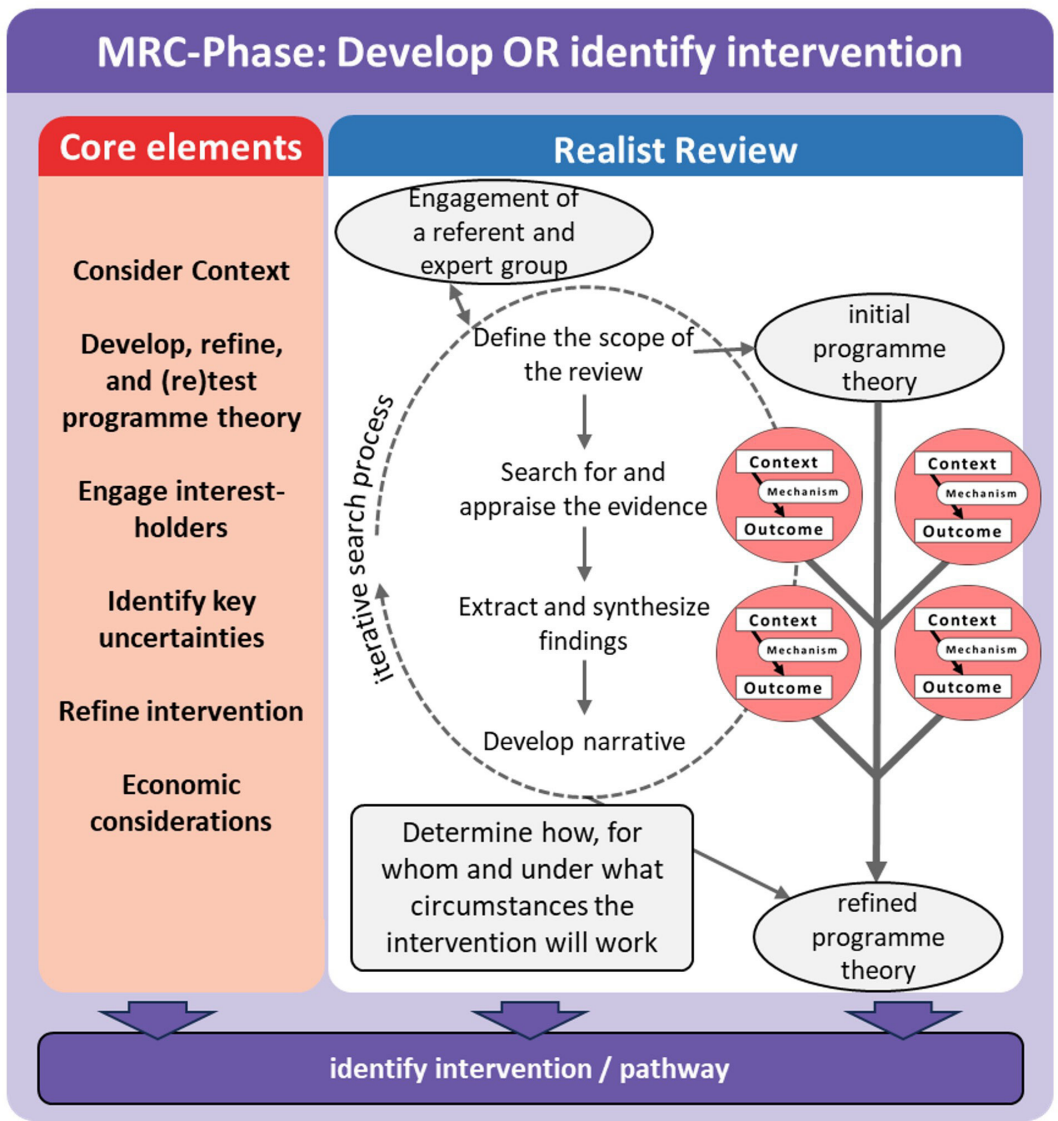
Every phase of the MRC framework should consider core elements (Compare Skivington *et al* 2021)^26^ and can be addressed through the realist review process to develop a refined programme theory and identify a possible intervention. The final programme theory and the underlying Context-Mechanism-Outcome model are an important result for the further phases of the MRC framework. Including interest holders as referent or expert groups into the review process as engagement methods is a promising aspect for better adherence in the later implementation processes. MRC, Medical Research Council.

By highlighting the importance of understanding mechanisms and contextual factors, the updated MRC framework supports the application of realist methodology.^27 28^ A realist review (RR) examines how an intervention works, for whom it is effective and under what conditions it achieves its goals.^29^,^31^ As an iterative theory-driven approach, the RR method can be responsive to local policy needs, while current evidence is considered. This directly addresses the underlying core elements of the MRC framework and the results of a RR are the key for intervention refinement for the phase ‘development or identification of interventions’ (see figure 1).

The RR approach is grounded in a programme theory based on the Context-Mechanism-Outcome (CMO) model, which describes how interventions generate outcomes within a specific context.^32^,^34^ Outcomes result from the interaction between context and mechanisms. Mechanisms emerge when an intervention introduces resources that, in a given context, influence participants’ reasoning or behaviour.^34^ Context includes material, social and situational factors (eg, organisational or interpersonal aspects) and is often dynamic and difficult to define.^33 35^ A key challenge is that context and mechanisms can overlap or evolve over time.^32^ A programme theory operationalises the CMO model by explaining why an intervention works, for whom it is effective and under what conditions. This theory can be mapped through a logic model.

### Aims and research questions

The aim of this RR is to develop a programme theory for a nurse-led CPW for preventing delirium in older adults aged 65 years and older in general hospital wards. This programme theory will seek to explain how, for whom and under what circumstances such a CPW may or may not be working.

The programme theory aims to address the following questions:

1. What are the essential components of a nurse-led CPW for delirium prevention in older adults in general hospital wards?
2. What contextual factors facilitate or hinder the implementation of a delirium prevention CPW in hospital settings?
3. Which strategies support the successful implementation of a delirium prevention CPW?
4. What are the key mechanisms through which the CPW is expected to produce its effects?
5. Which outcomes should be used to evaluate the effectiveness of a delirium prevention CPW?

## Methods and analysis

The methodology principles of Pawson *et al*^27^ have been widely applied in RRs, often with adjustments tailored to the specific research context.^36^,^38^ This review is structured following the adaptation by Rycroft-Malone *et al*^39^ for implementation research, which builds on the foundational work of Pawson *et al*.^29^ It comprises the following four key stages:

1. ‘Define the scope of the review’: this stage includes formulating the research question(s), clarifying the purpose of the review and developing an initial programme theory.
2. ‘Searching for and appraising the evidence’: in this phase, a purposive sampling strategy will be developed, followed by systematic searching and assessment of relevance and rigour of the included articles.
3. ‘Extracting and synthesising findings’: data will be extracted using a standardised tool, and findings will be synthesised to refine the initial programme theory.
4. ‘Developing a narrative synthesis’: this final stage involves engaging interest-holders and presenting findings, conclusions and practical recommendations.

The preparation of the study began in February 2025. The data search, screening and extraction are planned to start in August 2025, with final synthesis expected to be completed by November 2025. This protocol has been registered at Open Science Framework (https://doi.org/10.17605/OSF.IO/7EPTF) and is being reported in accordance with the Realist And MEta-narrative Evidence Syntheses: Evolving Standards (RAMESES) guideline.^30^

Interest-holder engagement is an integral part of this review process to frame the review question(s) for a successful implementation.^40^ A local reference and expert group will be actively involved to inform the review focus, interpret findings and guide potential implementation. This group will consist of relevant local interest-holders, like experts, decision-makers and knowledge users, representing the intended target audience of the intervention. The recruitment of the reference and expert group will be conducted through the review team’s existing professional networks. Experts are defined as nurses or physicians who have substantial clinical experience in the care of patients with delirium and/or have contributed to the field through publications or specialised training of delirium. Decision-makers refer to individuals who influence organisational procedures, such as nursing managers or clinical leads. Knowledge users are defined as professionals with technical and clinical expertise in the design and implementation of healthcare processes.

Involving such a reference group has shown beneficial effects on the quality and relevance of RRs.^40 41^ While the local reference and expert group supports contextual relevance during the theory-building phase, a broader interest-holder and patient engagement is essential for further phases of intervention development and conducting empirical studies.

### Patient and public involvement

Patients or members of the public will not be involved in the design, conduct, reporting or dissemination of this RR. However, broader patient and public involvement is planned for subsequent phases of intervention development and pilot testing of the CPW.

### Stage 1: define the scope of the review

First, the review team defined the scope and clarified the overall purpose of this project as a preparation step. To develop the scope, the authors conducted an exploratory and non-systematic literature search to gain a broad overview of delirium management in hospital settings worldwide with no limitation for publication date. The used keywords were a combination of terms related to delirium prevention, general hospital wards, guidelines and reviews. This includes academic sources (eg, peer-reviewed articles, dissertations, theses) accessed via general search engines (eg, Google Scholar, informal rapid search platforms), as well as grey literature (eg, government reports, clinical guidelines). Relevant publications had been flagged and retrieved for further examination.

In line with recommendations by Pawson *et al*,^29^ the review focuses on general hospital wards, literature from other hospital settings (eg, intensive care units or emergency departments) may be considered during the initial scoping phase if they offer transferable insights relevant to the development of the programme theory. However, only studies related to general hospital ward settings will be included in the formal review process. As this phase is not concerned with evaluating the effectiveness of interventions, the focus is on identifying underlying theories, assumptions and explanatory models, particularly regarding how interventions are expected to work and why they may fail in practice. The initial search was used to clarify the aim and the research questions.

In addition to the literature search, an interest-holder workshop will be conducted to incorporate the perspective of clinical practice. A local reference and expert group will be involved using the ‘3-blackboard method’, a structured consensus-building and brainstorming tool, to define the desired goals, describe the organisation of the care process and develop a list of relevant topics for the review process.^42^ The workshop is structured into three parts: (a) identifying goals of a delirium CPW; (b) defining key features of the desired care process and (c) outlining potential barriers and bottlenecks. Given that the resulting delirium CPW is intended to be piloted, the use of a developed method tailored for CPW design ensures that practical applicability is considered from the outset. The workshop will be documented in written form, and its outcomes will be used to validate findings from the review.

Drawing on insights from the exploratory literature search, internal group discussions and the interest-holder workshop, an initial CPW and initial programme theory will be developed. This theory will be structured using the CMO model and formulated as a series of if-then statements for example, “IF (Context), THEN (Mechanism) is triggered, LEADING TO (Outcome)”. Additionally, the programme theory will be represented graphically as a logic model. To facilitate its practical application, the Implementation Research Logic Model (IRLM) will be used to support the design and future implementation of the intervention.^43^

### Stage 2: search for and appraise the evidence

The search strategy and selection of search terms will be informed by the initial search, the workshop and with the support of a librarian. Keywords will include a combination of terms related to delirium, prevention, non-pharmacological interventions, CPWs, hospital settings and older adults aged 65 years or older. There will be no restrictions based on exclusion because of publication year or geographic location (see table 1). We will conduct a systematic search in the electronic databases Medline (via PubMed), CINAHL (via Ebsco) and Embase (via Elsevier).

**Table 1:** Search strategy and terms used in Medline (via PubMed)

Number	Explanation of the search terms	Search terms (combined with OR)
1	Population: persons aged 65 years and older	MeSH-terms: Aged; Geriatrics TITLE/ABSTRACT: old; older hospitalized patient*; older adult*; older inpatient*; elder*; aged; geriatric*; frail*; adult
2	Context: general hospital wards	MeSH-terms: Hospitals; Hospitalization TITLE/ABSTRACT: hospital*; general ward; normal ward; acute care; non ICU; non-ICU
3	Concept: delirium	MeSH-terms: Delirium; Confusion TITLE: Delirium; delir*; acute confusion; cognitive dysfunction; confus*; acute brain dysfunction
4	Concept: all interventions that can be part of a delirium prevention pathway	MeSH-terms: Prevention & control; patient care bundles TITLE/ABSTRACT: prevent*; monitor*; manag*; therap*; non-pharmacological; multicomponent; complex intervention; intervention*; programme*; training*; assess*
5	Concept: elements, that fit to: - the definition of clinical pathway based on the definition of Rotter *et al* or - includes the evaluation of the implementation process or includes parts of a programme theory or a logic model	MeSH-terms: Critical Pathways; Patient care planning; practice guidelines as topic; implementation science; quality improvement TEXT: Pathway*; protocol; care tracks; care plan; case management; critical care method; care map; care track; algorithm; care profile; program theor*; programme theor*; implementation; logic model, theory of change; theory of action; mechanism; context; guideline
6	#1 AND #2 AND #3 AND #4 AND #5	

In addition, this search will be supplemented by backward and forward citation searching and grey literature searches (including websites of relevant organisations, government statements, international guidelines and grey literature databases). As Pawson *et al* noted, up to 52% of relevant literature may be identified through snowballing techniques, with guidelines in particular often serving as sources.^29^ While RRs follow an iterative search process, search terms can be adapted with a new search cycle. Any changes will be addressed and detailed in the main manuscript. All included records will be collected with Zotero (V.7.0.13)^44^ and imported to Covidence^45^ for screening and data management. Duplicates will automatically be identified and removed using Covidence. Two review authors will independently screen titles, abstracts and full-text articles. If there are any disagreements between the two reviewers, a third member of the review team will be consulted. Manuscripts will be limited to those in English and German. We will include a wide range of study designs: quantitative, qualitative, mixed-methods and others that provide information on CMOs related to the initial programme theory will be included. All records will be appraised based on relevance and rigour (see table 2).

- Relevance: does the study contribute to the development or refinement of the programme theory?
- Rigour: are the methods used in the study credible and trustworthy?^30^

**Table 2:** Exclusion criteria for relevance and rigour

Exclusion criteria: relevance	Exclusion criteria: rigour
Only pharmacological intervention Only ICU or LTC, with no connection to general ward No relevance to IPT and/or delirium prevention for older adults (>65 years) No relevance for nurses (nurses cannot be part of the pathway)	No peer review Reports based on personal perspective (eg, opinion papers, case reports, short reports, pre-prints, protocols, conference abstracts)

ICU, intensive care unit; IPT, initial programme theory; LTC, long term care.

In cases where study results are published across multiple sources, the review team will identify and consolidate these sources accordingly. The screening process and selection process will be conducted independently by two reviewers. Reasons for exclusion will be documented and illustrated in a flow chart, following the RAMESES reporting standards.^30^

### Stage 3: extract and synthesise findings – extract data

**Figure 2:**
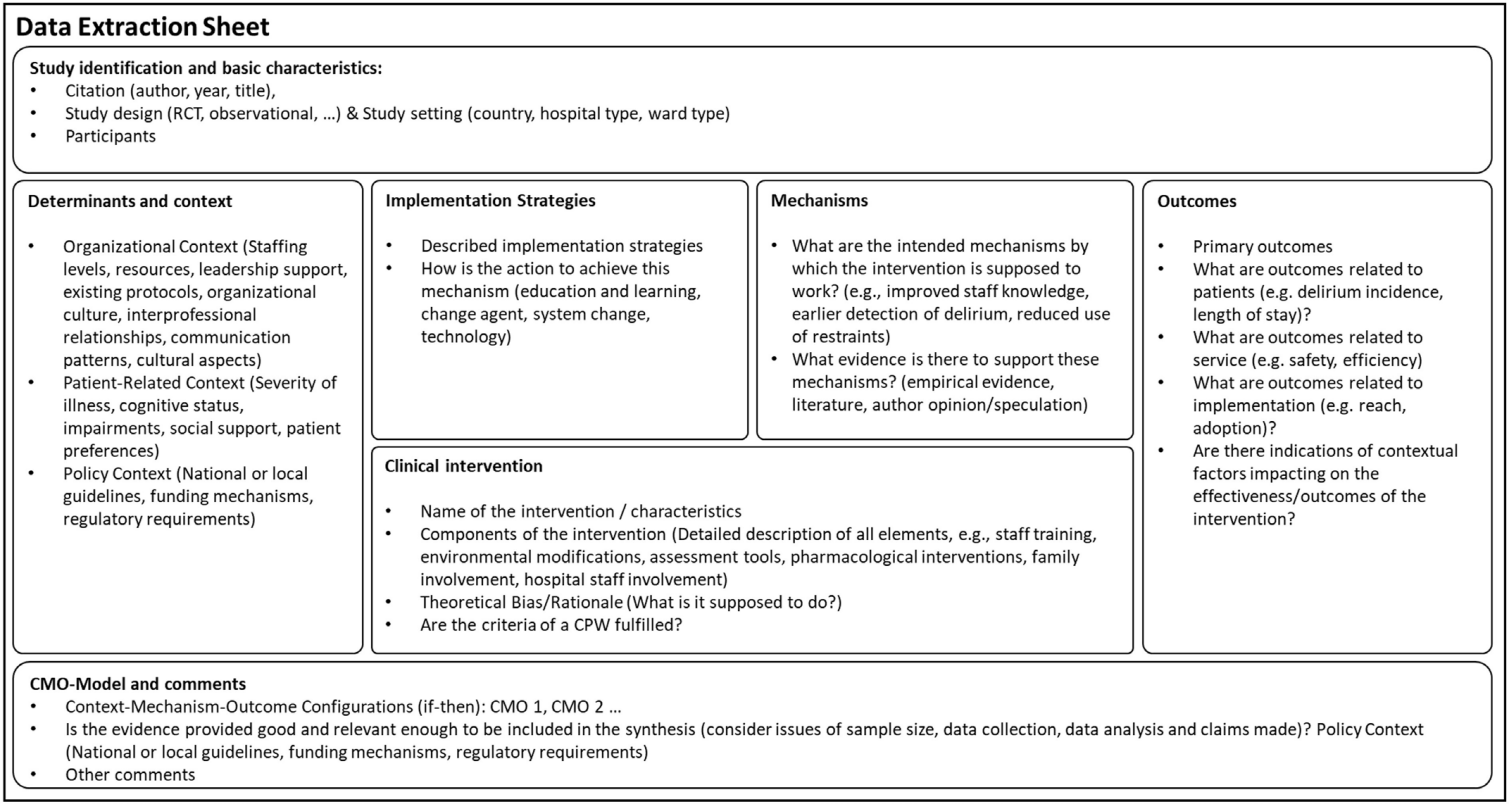
The data extraction sheet: the sheet is based on the Implementation Research Logic Model template for Comparative Implementation from Smith *et al*^43^ and is complemented with basic characteristics of the studies and Context-Mechanism-Outcome (CMO) configurations.

Following the recommendations,^39 43^ a standardised data extraction sheet was developed as part of the review preparation process and adapted to the specific aims of this review. The tool uses the IRLM as a structural template, with each section supplemented by predefined questions (see figure 2). Data will be synthesised using MAXQDA (V.2022),^46^ applying both deductive and inductive coding strategies. The research team developed a coding framework a priori, informed by the IRLM. This includes codes for ‘determinants and context’ and ‘implementation strategies’, drawing on the domains and constructs of the Consolidated Framework for Implementation Research^47^ and Expert Recommendations for Implementing Change.^48^ Reported outcomes will be categorised into patients-related, service-related or implementation-related outcomes. The identified CMO configurations will be represented and summarised using ‘if-then’ clauses like in stage 1. Inductive coding will be used to capture emerging themes and concepts not covered by the initial coding framework. One review author will extract and synthesise the findings, while a second author will validate and review the coding. If there is a difference of opinion, a third person will be contacted.

### Stage 4: develop narrative, draw conclusions and make recommendations

The refined programme theory will be presented to the expert panel, the reference group and key interest-holders on policy/organisational level for final recommendations and feedback. This will take place in the form of a follow-up workshop. The programme theory will be visualised using the IRLM framework, which will also be used to identify and map appropriate implementation strategies. This refined programme theory will inform subsequent phases of intervention development to establish a CPW intervention in the clinical practice in accordance with the MRC framework. In this later stage, the reference group will support the contextual adaptation and co-development of specific implementation strategies for a pilot study of the CPW in practice.

## Discussion

This publication aims to provide background information, methodological transparency and conceptual insights into the development of new clinical strategies for delirium prevention in terms of a CPW. In doing so, it may also support the implementation of additional interventions in clinical practice. In addition, the methodological approach of combining the MRC framework with RR methodology and operationalising both through the IRLM offers a novel perspective on complex intervention development with a strong orientation towards implementation and clinical impact.

Understanding how and why nurse-led delirium prevention interventions work can help to strengthen the role of nursing in clinical innovation, while simultaneously acknowledging the essential contribution of interprofessional teamwork. By making underlying mechanisms visible, this research supports interest-holders in grasping how interventions function within specific contexts. The resulting mid-range programme theory will inform next phases of the MRC framework and guide a pilot implementation study aimed at preventing delirium for older adults in general hospital wards.

## Ethics and dissemination

Since no data are collected as part of the review, ethical approval is not required. Findings will be disseminated through academic conferences and publication in a peer-reviewed journal.
