# Supplementary material for: Development of a nurse-led clinical pathway to prevent delirium in older adults in general hospital wards: a realist review protocol
Source: BMJ Open. 2025 Sep 15;15(9):e105801. doi: 10.1136/bmjopen-2025-105801 (PMC12439136; doi:10.1136/bmjopen-2025-105801)
Supplement: Supplementary file 1 [file bmjopen-2025-105801.reviewer_comments.pdf]

PEER REVIEW HISTORY

BMJ Open publishes all reviews undertaken for accepted manuscripts. Reviewers are asked to complete a checklist review form and are provided with free text boxes to elaborate on their assessment. These free text comments are reproduced below.

ARTICLE DETAILS

Title (Provisional)

Development of a nurse-led clinical pathway to prevent delirium in older adults in general hospital wards: a realist review protocol

Authors

Hoch, Jonas; MÜLLER, Martin; Unger, Inga; Mitzkat, Anika; Denninger, Natascha-Elisabeth

---

VERSION 1 - REVIEW

**Reviewer**	**1**
**Name**	**Jerofke-Owen, Teresa**
**Affiliation**	**Marquette University - College of Nursing**
**Date**	**09-Jul-2025**
**COI**	**None**

Thank you for the opportunity to review this protocol. The authors address an important and timely topic. There are some areas that would benefit from further clarification. My comments are listed below:

Abstract: RAMESES is a set of reporting and quality standards but is not a realist review methodology – would cite pawson/Rycroft malone's method and those steps in the abstract. You can state that you will follow RAMESES guidelines to ensure thorough reporting, but that is not your method. Also would recommend defining what you mean by older adults.

The first bullet under strengths and limitations is a bold statement. I would lessen the language and state that the initial programme theory can help guide the development of future interventions.

Introduction: Again please refine "older adults". I think you are missing some key references that actually examine nurse-led interventions for delirium. For instance – this is a new reference that should be part of this manuscript: Wang, J., Lu, Y., Chen, X., & Wu, Y. (2024). Effectiveness of nurse-led non-pharmacological interventions on outcomes of delirium in adults: A meta-analysis of randomized controlled trials. Worldviews on Evidence-Based Nursing, 21(5), 514–527. https://doi.org/10.1111/wvn.12739

Please be more specific about who the stakeholders are and how you will recruit them to be a part of this project. Who are the local stakeholders, decision-makers, and knowledge users you speak of? Would recommend you have a health librarian on your team to ensure a thorough search. I am also confused how you are talking about defining the scope of the review in the future but your aim and research questions have already been established? Shouldn't this be part of stage 1? Please clarify.

Would like some clarification about how a data extraction sheet is already developed to extract data for the final programme theory when the initial programme theory has not even been established yet? It seems like much of the process of this realist review has been established even though during stage 1 the authors discuss a stakeholder group and the role it will play in planning this realist review.

Please be more detailed about how you will determine inclusion/exclusion criteria for your search. It isn't clear what you mean by stating "this will be informed by the initial program theory"? What search strategy will you use for stage 1 "exploratory literature search" ? Please be more specific – are you looking worldwide? Are you searching certain publication years?

Thank you

**Reviewer**	**2**
**Name**	**Pereira Gray, Denis**
**Affiliation**	**St Leonard's Research Practice**
**Date**	**16-Jul-2025**
**COI**	**None**

STRENGTHS

- **Delirium is a serious condition**
- **It is highly disturbing for patients**
- **It takes much extra time and work for staff**
- **Prevention is a high priority**
- **Nurses are well placed to contribute**
- **A protocol for a nurse pathway is logical and welcome**
- **The protocol has been registered, which is good practice.**

LIMITATIONS

- Since so much depends upon the search process it would be worth considering including the proposed initial search terms

- There has been important nursing research by Linda Aiken showing that the level of nurse staffing and the proportion of nurses with full qualifications is strongly associated with outcomes so these factors should be included.
  Aiken, Linda H., Douglas Sloane, Peter Griffiths, Anne Marie Rafferty, Luk Bruyneel, Matthew McHugh, Claudia B. Maier et al. "Nursing skill mix in European hospitals: cross-sectional study of the association with mortality, patient ratings, and quality of care." *BMJ quality & safety* 26, no. 7 (2017): 559-568.
- In line 88 it is stated that the proposed pathway is to be 'multidisciplinary , ' but there are no other references to other professions. This needs clarification. For example, the prescription of drugs by doctors to patients with dementia has big influence on delirium (Delgado J *et al* (2022) ; declare an interest.
  Delgado, J., Evans, P.H., Pereira Gray, D., Sidaway-Lee, K., Allan, L., Clare, L., Ballard, C., Masoli, J., Valderas, J.M. and Melzer, D., (2022P Continuity of GP care for patients with dementia: impact on prescribing and the health of patients. *British Journal of General Practice*
- The editorial guideline states that dates should be included and they are not.
- Whilst is not a requirement that patients should be involved it is good practice to so.
- The title refers to general hospital wards, but the text refs to other wards. This needs clarification. Either the title needs to change or the text and you readers need to now what is intended.

Use of English

Programme is spelt the American way.

Line 214 Better is:

Manuscripts will be limited to those in English and German.

CONCLUSION

Advise accepting for publication after tidying up the above points.

Professor Sir Denis Pereira Gray OBE HonDSc FRCP FRCGP FMedSci

Emeritus Professor, University of Exeter, UK

VERSION 1 - AUTHOR RESPONSE

<u>**Feedback Reviewer 1**</u>	<u>**Authors´ response**</u>
REVIEWER 1, COMMENT 1:	Dear Dr. Jerofke-Owen,

Thank you for the opportunity to review this protocol. The authors address an important and timely topic. There are some areas that would benefit from further clarification. My comments are listed below:<br><br>Abstract: RAMESES is a set of reporting and quality standards but is not a realist review methodology – would cite pawson/Rycroft malone's method and those steps in the abstract. You can state that you will follow RAMESES guidelines to ensure thorough reporting, but that is not your method.	thank you for your helpful comments and for highlighting the distinction between realist methodology and reporting standards. Your review was very helpful to improve our work, and we hope we could improve it for the second throughput.<br><br>---<br><br>We have clarified this in the abstract. We now state that the review is based on the methodological work of Pawson and Rycroft-Malone, and that reporting will follow the RAMESES guidelines.<br><br>**Changes:**<br>Text position: "ABSTRACT", page 2:<br>**Methods and analysis**<br>The realist review is based on the methodical framework developed by Pawson et al. and further adapted by Rycroft-Malone et al. and the reporting will follow the RAMESES (Realist And MEta-narrative Evidence Syntheses: Evolving Standards) guidelines.
REVIEWER 1, COMMENT 2:<br>Also would recommend defining what you mean by older adults.	We have also defined the term "older adults" more clearly as persons aged 65 years or older, both in the abstract and throughout the manuscript.<br><br>**Changes:**<br>Text position: "ABSTRACT" page 2:<br><br>**Introduction**<br>[…]<br>The aim of this realist review is to develop a programme theory for a nurse-led clinical pathway to prevent delirium in older adults aged 65 years or older in general hospital wards and to identify strategies to support its effective implementation.<br><br>Text position: "Aims and research question" page 6:<br><br>The aim of this realist review is to develop a programme theory for a nurse-led clinical pathway for preventing delirium in older adults aged 65 and older in general hospital wards. This programme theory will seek to explain how, for whom and under what circumstances such a CPW may or may not be working.
REVIEWER 1, COMMENT 3:<br>The first bullet under strengths and limitations is a bold statement. I would lessen the language and state that the initial programme	We lessen the language and rewrite the first bullet point.<br><br>**Changes:**<br>See also Editor, Comment 1

theory can help guide the development of future interventions.	Text position: *"STRENGTHS AND LIMITATIONS OF THIS STUDY"*, page 3<br><br>• The initial programme theory developed in this review may help to guide the design of future complex interventions for delirium prevention.<br>• The programme theory will be developed through iterative synthesis of diverse data sources.<br>• Interest holder engagement is integrated throughout the review to inform scope and interpretation.<br>• Quality appraisal focuses on relevance and rigor rather than risk of bias, which may affect comparability with traditional systematic reviews.
REVIEWER 1, COMMENT 4:<br><br>Introduction: Again please refine "older adults".	We clarify the term "older adults", see the answer above.
REVIEWER 1, COMMENT 5:<br>I think you are missing some key references that actually examine nurse-led interventions for delirium. For instance – this is a new reference that should be part of this manuscript: Wang, J., Lu, Y., Chen, X., & Wu, Y. (2024). Effectiveness of nurse-led non-pharmacological interventions on outcomes of delirium in adults: A meta-analysis of randomized controlled trials. Worldviews on Evidence-Based Nursing, 21(5), 514–527. https://doi.org/10.1111/wvn.12739	We also include this reference now. This is a useful suggestion; we appreciate this.<br><br>**Changes:**<br>Text position: "INTRODUCTION", page 4:<br>In contrast, evidence highlighted that multi-component, non-pharmacological interventions, targeting various risk factors for delirium like re-orientation, cognitive stimulation and sleep hygiene, were associated with a 27% to 54% reduction in delirium incidence among older people in general hospital wards [11–13]. There is also evidence that nursing skill influences the quality of care, particularly through nurse-led non-pharmacological delirium interventions that are associated with reduced mortality [14,15].
REVIEWER 1, COMMENT 6:<br><br>Please be more specific about who the stakeholders are and how you will recruit them to be a part of this project. Who are the local stakeholders, decision-makers, and knowledge users you speak of?	The acquisition of the reference and expert group is realized through personal networking of the review team. Experts are defined as nurses or physicians with previous publications in the field of delirium, or who have received special training for delirium, and have professional experience in the care of patients with delirium. Stakeholders and decision-makers are defined as people with an impact on organizational procedures, like nursing managers. While knowledge users include persons with technical and clinical knowledge about designing and implementing processes.<br>Also, we changed stakeholder to "interest-holder" see the following additional comment.<br><br>**Changes:**

	Text position: “METHODS AND ANALYSIS”, page 7:<br><br>Interest-holder engagement is an integral part of this review process to frame the review question(s) for a successful implementation [40]. A local reference and expert group will be actively involved to inform the review focus, interpret findings, and guide potential implementation. This group will consist of relevant local interest-holders, like experts, decision-makers, and knowledge users, representing the intended target audience of the intervention. The recruitment of the reference and expert group will be conducted through the review team´s existing professional networks. Experts are defined as nurses or physicians who have substantial clinical experience in the care of patients with delirium and/or have contributed to the field through publications or specialized training of delirium. Decision-makers refer to individuals who influence organizational procedures, such as nursing managers or clinical leads. Knowledge users are defined as professionals with technical and clinical expertise in the design and implementation of healthcare processes.
**Additional comment**	In order to be even more sensitive to language, we have also decided to follow the statement of the MuSE Consortium and change the term “stakeholder” to “interest-holder” to convey the intended meaning without any negative connotations.<br><br>Akl, E. A., Khabsa, J., Petkovic, J., Magwood, O., Lytvyn, L., Motilall, A., ... & Concannon, T. W. (2024). “Interest-holders”: A new term to replace “stakeholders” in the context of health research and policy. Cochrane Evidence Synthesis and Methods, 2(11), e70007. https://doi.org/10.1002/cesm.70007
REVIEWER 1, COMMENT 7:<br>Would recommend you have a health librarian on your team to ensure a thorough search.	We decided to have a consultation with a librarian and added the search string as a new table.<br><br>**Changes:**<br>EDITOR 1, COMMENT 3.<br><br>Text position: *“Stage 2: Search for and appraise the evidence”* page 8:<br>The search strategy and selection of search terms will be informed by the initial search, the workshop and with the support of a librarian. Keywords will include a combination of terms related to delirium, prevention, non-pharmacological interventions, clinical pathways, hospital settings, and older adults aged 65 years or older. There will be no restrictions based on exclusion because of publication year or geographic location (see table 1).<br><br>Table 1. Search strategy and terms used in Medline (via PubMed).

	Text position: "Stage 2: Search for and appraise the evidence" page 11:<br>Table 2. Exclusion criteria for relevance and rigor.
REVIEWER 1, COMMENT 8:<br>I am also confused how you are talking about defining the scope of the review in the future but your aim and research questions have already been established? Shouldn't this be part of stage 1? Please clarify.	We agree that defining the scope of the review is formally part of Stage 1 of the realist review process. However, as part of the protocol preparation, some steps of this stage have already been completed.<br>Specifically, we conducted an initial exploratory and non-systematic literature search to inform the development of the review scope and to draft preliminary research questions. In July, we also completed the stakeholder workshop to further refine the scope and direction of the review.<br>As per *BMJ Open* guidance for study protocols, it is acceptable to present baseline data used in planning the review, as long as no review results or conclusions are reported. Therefore, the results of the initial program theory development will not be included in the protocol, but will be presented in the main review manuscript.<br><br>**Changes:**<br>Text position: "Stage 1: Define the scope of the review" page 8:<br>First, the review team defined the scope and clarified the overall purpose of this project as a preparation step. To develop the scope, the authors conducted an exploratory and non-systematic literature search to gain a broad overview of delirium management in hospital settings worldwide with no limitation for publication date. The used keywords were a combination of terms related to delirium prevention, general hospital wards, guidelines, and reviews. This includes academic sources (e.g. peer-reviewed articles, dissertations, theses) accessed via general search engines (e.g. Google scholar, informal rapid search platforms), as well as grey literature (e.g. government reports, clinical guidelines). Relevant publications had been flagged and retrieved for further examination.<br>In line with recommendations by Pawson et al. [29], the review focuses on general hospital wards, literature from other hospital settings (e.g. intensive care units or emergency departments) may be considered during the initial scoping phase if they offer transferable insights relevant to the development of the programme theory. However, only studies related to general hospital ward settings will be included in the formal review process. As this phase is not concerned with evaluating the effectiveness of interventions, the focus is on identifying underlying theories, assumptions, and explanatory models, particularly regarding how interventions are expected to work and why they may fail in practice. The initial search was used to clarify the aim and the research questions.

REVIEWER 1, COMMENT 9:<br><br>Would like some clarification about how a data extraction sheet is already developed to extract data for the final programme theory when the initial programme theory has not even been established yet? It seems like much of the process of this realist review has been established even though during stage 1 the authors discuss a stakeholder group and the role it will play in planning this realist review.	The data extraction tool was developed during the preparatory phase of the review and is based on established methodological frameworks, including the work of Pawson, the adaptation by Rycroft-Malone, and particularly the Implementation Research Logic Model (IRLM) by Smith et al.<br><br>Importantly, the tool was designed prior to the development of an initial program theory, and does not rely on specific theoretical assumptions. It is structured to capture relevant data on context, mechanisms, and outcomes in a way that is flexible and adaptable as the program theory emerges.<br><br>Our intention was to create a transparent and reproducible extraction framework that can serve as a generic template, not only for this review, but also for similar realist-informed implementation studies.
REVIEWER 1, COMMENT 10:<br><br>Please be more detailed about how you will determine inclusion/exclusion criteria for your search. It isn't clear what you mean by stating "this will be informed by the initial program theory"? What search strategy will you use for stage 1 "exploratory literature search"? Please be more specific – are you looking worldwide? Are you searching certain publication years?<br>Thank you	In your comment 7 and the comment 3 of the editor we addressed this issue. With the help of a medical librarian, we optimized the initial search strategy in the manuscript. We also added two tables, table 2 addresses the exclusion criteria for the screening process, while in table 1 there is an explanation of the search terms, and why we include them.<br><br>**<u>Changes:</u>**<br>EDITOR 1, COMMENT 3.

**<u>Feedback Reviewer 2</u>**	**<u>Authors´ response</u>**
REVIEWER 2, COMMENT 1:<br><br>Since so much depends upon the search process it would be worth considering including the proposed initial search terms	Dear Prof. Pereira Gray,<br>we greatly appreciate this review responses due to the appreciative and very helpful additions. This led to a good discussion within our team, and we hope to have refined our manuscript now.<br>---<br>We decided to have a consultation with a librarian and added the search string as a new table (see also EDITOR 1, COMMENT 3, REVIEWER 1 COMMENT 7 & 8 & 10).<br><br>**<u>Changes:</u>**<br>EDITOR 1, COMMENT 3.<br><br>Text position: "Stage 1: Define the scope of the review" page 8:

	First, the review team defined the scope and clarified the overall purpose of this project as a preparation step. To develop the scope, the authors conducted an exploratory and non-systematic literature search to gain a broad overview of delirium management in hospital settings worldwide with no limitation for publication date. The used keywords were a combination of terms related to delirium prevention, general hospital wards, guidelines, and reviews. This includes academic sources (e.g. peer-reviewed articles, dissertations, theses) accessed via general search engines (e.g. Google scholar, informal rapid search platforms), as well as grey literature (e.g. government reports, clinical guidelines). Relevant publications had been flagged and retrieved for further examination.<br>In line with recommendations by Pawson et al. [29], the review focuses on general hospital wards, literature from other hospital settings (e.g. intensive care units or emergency departments) may be considered during the initial scoping phase if they offer transferable insights relevant to the development of the programme theory. However, only studies related to general hospital ward settings will be included in the formal review process. As this phase is not concerned with evaluating the effectiveness of interventions, the focus is on identifying underlying theories, assumptions, and explanatory models, particularly regarding how interventions are expected to work and why they may fail in practice. The initial search was used to clarify the aim and the research questions.<br><br>Text position: *"Stage 2: Search for and appraise the evidence"* page 8:<br>The search strategy and selection of search terms will be informed by the initial search, the workshop and with the support of a librarian. Keywords will include a combination of terms related to delirium, prevention, non-pharmacological interventions, clinical pathways, hospital settings, and older adults aged 65 years or older. There will be no restrictions based on exclusion because of publication year or geographic location (see table 1).<br><br>Table 1. Search strategy and terms used in Medline (via PubMed).<br><br>Text position: "Stage 2: Search for and appraise the evidence" page 11:<br>Table 2. Exclusion criteria for relevance and rigor.
There has been important nursing research by Linda Aiken showing that the level of nurse staffing and the proportion of nurses with full qualifications is strongly associated with outcomes so these factors should be included.	Thank you for suggesting this reference; we included this one in our introduction.<br><br>**Changes:**<br>Text position: "INTRODUCTION" page4

Aiken, Linda H., Douglas Sloane, Peter Griffiths, Anne Marie Rafferty,<br>Luk Bruyneel, Matthew McHugh, Claudia B. Maier et al. "Nursing skill mix in European hospitals: cross-sectional study of the association with mortality, patient ratings, and quality of care." BMJ quality & safety 26, no. 7 (2017): 559-568.	There is also evidence that nursing skill influences the quality of care, particularly through nurse-led non-pharmacological delirium interventions that are associated with reduced mortality [14,15].<br>[…]<br>Despite the integral collaboration of different professions and disciplines, a delirium pathway should be nurse-led, since the recognition of patients' immediate and often subtle behavioural or cognitive changes requires continuous presence and clinical judgment. Nurses are uniquely positioned to identify the fluctuating course of delirium through systematic observation, bedside assessment, and the integration of contextual and biographical knowledge. These competencies are essential for timely detection, escalation, and intervention core elements of effective delirium management. Therefore, the leadership of a delirium clinical pathway should be rooted in nursing expertise, while embedded in an interprofessional framework, e.g. if aspects like deprescribing of inappropriate medication or specific mobility trainings might be included [14,23].
REVIEWER 2, COMMENT 2:<br><br>In line 88 it is stated that the proposed pathway is to be "multidisciplinary", but there are no other references to other professions. This needs clarification. For example, the prescription of drugs by doctors to patients with dementia has big influence on delirium (Delgado J et al (2022) ; declare an interest. Delgado, J., Evans, P.H., Pereira Gray, D., Sidaway-Lee, K., Allan, L., Clare, L., Ballard, C., Masoli, J., Valderas, J.M. and Melzer, D., 2022P Continuity of GP care for patients with dementia: impact on prescribing and the health of patients. British Journal of General Practice	Thank you for suggesting this reference; we included this one in our introduction.<br><br>**<u>Changes:</u>**<br>Text position: "INTRODUCTION" page4<br>[…]<br>Despite the integral collaboration of different professions and disciplines, a delirium pathway should be nurse-led, since the recognition of patients' immediate and often subtle behavioural or cognitive changes requires continuous presence and clinical judgment. Nurses are uniquely positioned to identify the fluctuating course of delirium through systematic observation, bedside assessment, and the integration of contextual and biographical knowledge. These competencies are essential for timely detection, escalation, and intervention core elements of effective delirium management. Therefore, the leadership of a delirium clinical pathway should be rooted in nursing expertise, while embedded in an interprofessional framework, e.g. if aspects like deprescribing of inappropriate medication or specific mobility trainings might be included [14,23].
REVIEWER 2, COMMENT 3:<br><br>The editorial guideline states that dates should be included and they are not.	We added this information.<br>**<u>Changes:</u>**<br>See EDITOR 1, COMMENT 2.
REVIEWER 2, COMMENT 4:<br><br>Whilst is not a requirement that patients should be involved it is good practice to so.	We acknowledge that patient involvement is not mandatory but considered good practice. As described in the methods section, patients and the public were not involved in this realist review phase. However, we plan to include patient and public involvement in the subsequent phases of intervention development and pilot testing (please see also Editor, comment 4).

	**Changes:**<br>Text position: "METHODS AND ANALYSIS" page 7:<br>**Patient and public involvement**<br>Patients or members of public will not be involved in the design, conduct, reporting, or dissemination of this realist review. However, broader patient and public involvement is planned for subsequent phases of intervention development and pilot testing of the clinical pathway.
REVIEWER 2, COMMENT 5:<br><br>The title refers to general hospital wards, but the text refs to other wards. This needs clarification. Either the title needs to change or the text and you readers need to now what is intended.	Thank you for this helpful comment. We confirm that the focus of this realist review is on general hospital wards, as stated in the title. To avoid confusion, we have revised the relevant section in the methods to clarify that literature from other hospital settings (e.g., ICU or emergency departments) may be considered during the initial scoping phase only if it provides transferable insights to inform the program theory. However, the inclusion criteria for the review itself are limited strictly to general hospital ward settings.<br><br>**Changes:**<br>Text position: "Stage 1: Define the scope of the review:", page 8:<br><br>In line with recommendations by Pawson et al. [29], the review focuses on general hospital wards, literature from other hospital settings (e.g. intensive care units or emergency departments) may be considered during the initial scoping phase if they offer transferable insights relevant to the development of the programme theory. However, only studies related to general hospital ward settings will be included in the formal review process.
REVIEWER 2, COMMENT 6:<br><br>Use of English<br>Programme is spelt the American way.	We were aware, that "program" is the American spelling, we use this as basic setting in our grammar check system. We agree that, if we publish in BMJ Open, we should use UK-settings, so we change the wording *program* to *programme*. This includes figure 1.<br><br>**Changes:**<br>We don't link all changes here, there was more than 30 changes (program to programme).
REVIEWER 2, COMMENT 7:<br><br>Line 214 Better is:<br>Manuscripts will be limited to those in English and German	We have adapted the proposal.<br><br>**Changes:**<br>Text position: "Stage 2: Search for and appraise the evidence:", page 10:<br><br>If there are any disagreements between the two reviewers, a third member of the review team will be consulted. Manuscripts will be limited to those in English and German.
